# Supplementary material for: Can parasites halt the invader? Mermithid nematodes parasitizing the yellow-legged Asian hornet in France
Source: PeerJ. 2015 May 21;3:e947. doi: 10.7717/peerj.947 (PMC4451032; doi:10.7717/peerj.947)
Supplement: Supplemental Information 1 — French version of ”Can parasites halt the invader? Mermithid nematodes parasitizing the yellow-legged Asian hornet in France”. [file peerj-03-947-s001.pdf]

Traduction intégrale en français de l'article :

Can parasites halt the invader? Mermithid nematodes parasitizing the yellow-legged Asian hornet in France

Claire Villemant, Dario Zuccon, Quentin Rome, Franck Muller, George O. Poinar Jr., Jean-Lou Justine

Publié dans PeerJ, 2015
DOI 10.7717/peerj.947

Des parasites peuvent-ils stopper l'invasion ? Des nématodes mermithidés parasitent le frelon asiatique à pattes jaunes en France

Claire Villemant [1], Dario Zuccon [2], Quentin Rome [1], Franck Muller [1], George O. Poinar Jr. [3], Jean-Lou Justine [1]

[1] *Institut de Systématique, Évolution, Biodiversité, ISYEB, UMR 7205 – CNRS, MNHN, UPMC, EPHE, Muséum National d'Histoire Naturelle, Sorbonne Universités, 57 rue Cuvier, CP50, 75231 Paris cedex 05, France*

[2] *Service de Systématique moléculaire, UMS 2700 CNRS, Muséum National d'Histoire Naturelle, 57 rue Cuvier, 75231 Paris cedex 05, France*

[3] *Department of Integrative Biology, Oregon State University, Corvallis, OR 97331, USA*

Traduction : C. Villemant

Contact: Claire Villemant

ISYEB, Institut de Systématique, Évolution, Biodiversité
(UMR7205 CNRS, EPHE, MNHN, UPMC)
Muséum National d'Histoire Naturelle
CP 50, 45 rue Buffon, 75231 Paris cedex 05, France

Telephone: 01 40 79 38 41 (+33 1 40 79 38 41)
e-mail: villeman@mnhn.fr

RESUME

Depuis son introduction en France il y a 10 ans, le frelon asiatique à pattes jaunes *Vespa velutina* s'est rapidement propagé dans les pays voisins (Espagne, Portugal, Belgique, Italie et Allemagne). Ce prédateur qui chasse les abeilles en vol devant les ruches constitue une nouvelle menace pour l'apiculture européenne.

Si les espèces exotiques sont souvent introduites dans un nouvel environnement sans leurs ennemis naturels, ce qui favorise leur multiplication, elles peuvent aussi subir l'attaque de nouveaux ennemis naturels d'origine locale.

Trois jeunes adultes de la famille des mermithidés ont été obtenus en 2012 d'adultes de *V. velutina*, dans deux localités françaises. Ces vers parasites sont les seuls observés jusqu'à ce jour en Europe sur le frelon invasif, malgré le grand nombre de nids détruits chaque année et une étude récente portant sur de plus de 30 000 frelons adultes. Ceci laisse à penser que l'infection de *V. velutina* par ces nématodes demeure exceptionnelle.

Les spécimens ont été rattachés au genre *Pheromermis* par leurs caractères morphologiques mais seulement à la famille des Mermithidae par les données moléculaires (séquences du gène 18S), du fait de l'absence de séquences de *Pheromermis* spp. dans GenBank. Les vers appartiennent probablement à l'espèce *Pheromermis vesparum*, un parasitoïde de guêpes sociales en Europe. Ce nématode est le deuxième ennemi autochtone de *Vespa velutina* observé en France, après une mouche Conopidae dont les larves se développent en parasitoïdes internes des adultes de guêpes sociales et de bourdons. Cet article démontre que le nématode parasite est nécessairement d'origine locale et que son impact sur la survie des colonies de guêpes sociales demeure très limité. Nous présentons également des arguments expliquant pourquoi ces parasitoïdes (mermithidés et conopidés) n'ont aucune chance d'entraver l'invasion du frelon asiatique et ne peuvent donc être des auxiliaires efficaces pour un programme de lutte biologique contre cette espèce envahissante.

INTRODUCTION

L'introduction récente du frelon asiatique *Vespa velutina* en France constitue le premier cas d'invasion d'un Vespidae exotique en Europe (Rasplus et al., 2010 ; Beggs et al., 2011). Cette espèce envahissante préoccupe fortement les apiculteurs et les pouvoirs publics à cause de sa multiplication rapide et de son impact sur l'apiculture (Perrard et al., 2009) ; ce frelon est non seulement un prédateur d'abeilles mais sa présence en vol stationnaire devant les ruches perturbe l'activité des colonies (Rortais et al., 2010 ; Monceau et al., 2013 ; Arca et al., 2014). L'espèce a été observée pour la première fois en 2004 dans le sud-ouest de la France (Villemant et al., 2011) et s'est propagée depuis à travers 67 départements français (soit environ 360 000 km² ; Fig. 1) (Rome et al., 2013 ; INPN, 2015) ; elle est parvenue en Espagne en 2010, au Portugal et en Belgique en 2011 (Rome et al., 2013), en Italie en 2012 (Demichelis et al., 2014) et en Allemagne en 2014 (R. Witt. Pers. comm., 2014). Elle risque de se propager dans toute l'Europe (Villemant et al., 2011) et les récents scénarios de changement climatique font craindre une expansion future encore plus rapide que celle des dix dernières années (Barbet-Massin et al., 2013).

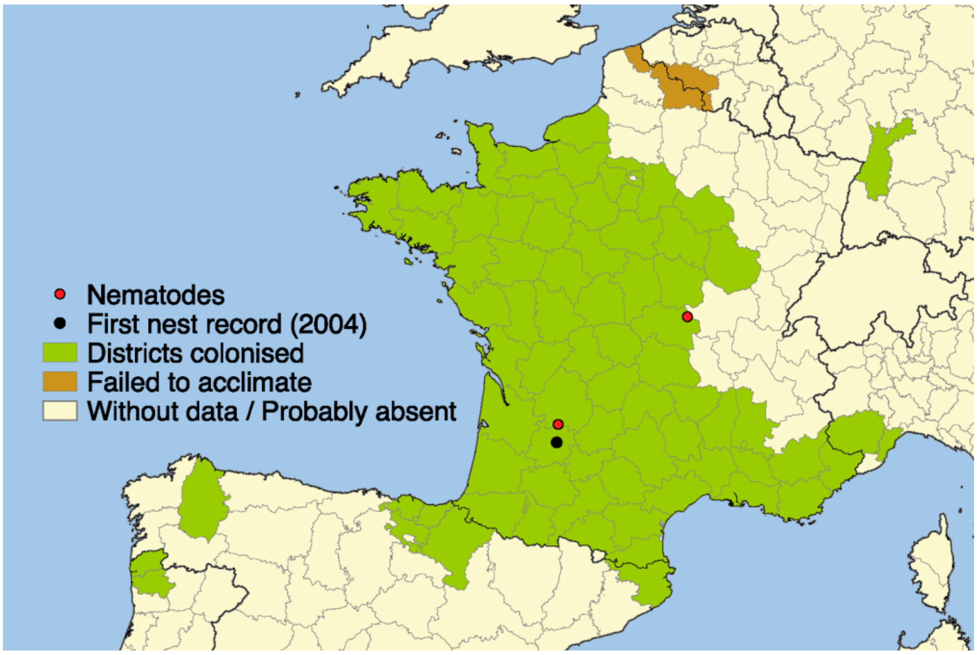

Figure 1. Cartographie de l'invasion du frelon asiatique à pattes jaunes *Vespa velutina* en Europe en 2014. Cercle noir: premier signalement de *V. velutina* en Europe. Cercles rouges: localités où les nématodes ont été trouvés.

De multiples facteurs biotiques, comme les ressources nutritives, la compétition interspécifique ou les ennemis naturels peuvent intervenir, indépendamment ou non, sur la démographie d'une espèce envahissante et influencer ainsi son devenir. Beaucoup d'espèces exotiques sont introduites dans un nouvel environnement sans leurs ennemis naturels, ce qui accroit leurs capacités de reproduction et de compétition (Holway, Suarez & Case, 1998 ; Colautti et al., 2004 ; Torchin & Mitchell, 2004 ; Lee & Klasing, 2004 ; Roy et al., 2011). Mais de nouveaux ennemis naturels d'origine autochtone peuvent aussi être recrutés dans

l'aire d'invasion et, en se multipliant sur ce nouvel hôte, affecter non seulement les populations de l'envahisseur mais aussi celles des hôtes autochtones (Prenter et al., 2004 ; Girardoz, Kenis & Quicke, 2006 ; Dunn, 2009 ; Kenis et al., 2009 ; Péré et al, 2011).

Le premier ennemi autochtone de *Vespa velutina* signalé en Europe est une mouche de la famille des Conopidae, dont les larves se développent en parasitoïdes internes de guêpes et de bourdons adultes (Darrouzet, Gévar & Dupont, 2014). Nous signalons ici la découverte en France d'un nouveau parasite, un nématode Mermithidae du genre *Pheromermis* qui a été obtenu en 2012 d'adultes de *V. velutina* capturés dans deux localités relativement éloignées l'une de l'autre. A notre connaissance, aucun autre nématode parasite de *V. velutina* n'a été jusqu'à ce jour signalé en Europe.

Dans cet article, nous nous interrogeons sur les potentialités de ces parasites à limiter la multiplication du frelon asiatique et sur leur éventuelle utilisation comme auxiliaires dans des programmes de lutte biologique contre cette espèce envahissante.

MATERIELS ET METHODES

Origine des spécimens

La progression de l'invasion de *Vespa velutina* (Figure 1) est suivie depuis 2006 grâce à une base de données en ligne sur la biodiversité gérée par le Muséum National d'Histoire Naturelle (MNHN) et régulièrement mise à jour par l'un de nous, QR (Rome et al., 2013 ; INPN, 2015). Les données de plus de 7 000 nids ont été enregistrées entre 2006 et 2014. Les nids sont principalement observés en automne, après la chute des feuilles, lorsque les colonies ont atteint leur maturité et contiennent de plusieurs centaines àdeux mille frelons adultes (Rome et al., 2015). Ce suivi a permis d'acquérir beaucoup d'informations utiles sur la localisation des nids et la progression de l'invasion, mais n'a fourni aucune donnée sur la présence de parasites au sein des colonies. Cependant, une étude récente sur l'évolution saisonnière des colonies de *V. velutina* nous a conduit entre 2007 et 2011 à disséquer 77 nids, provenant de quatre départements français (Dordogne et Gironde principalement) envahis depuis longtemps (Rome et al., 2015). Ces nids, conservés au congélateur à -25°C, ont été décongelés pour la dissection et quelques 33 000 frelons adultes en ont été extraits pour être triés et pesés. Au cours de cette manipulation, aucun individu susceptible d'être parasité par un nématode, c'est-à-dire ayant l'abdomen distendu, flasque ou éclaté, n'a été observé. Aucun frelon n'a été disséqué mais si l'un d'entre eux avait été infecté, la présence d'un nématode n'aurait pu nous échapper. En effet, à la fin de son développement le nématode parasite est pelotonné à l'intérieur de l'abdomen de son hôte, dont les segments se détachent facilement les uns des autres après avoir subi une congélation suivie d'une décongélation. Cela a été le cas pour le seul frelon parasité que nous ayons pu observer et qui contenait une nymphe de Conopidae, restée non identifiée (Villemant et al., 2008).

La présence de frelons parasités par des Mermithidae a été signalée par des particuliers à deux reprises : en novembre 2012, un nématode a été observé lors de la dissection de dix frelons adultes issus d'un nid détruit à Dompierre-sur-Besbre, Allier (Ph. Noireterre. comm. pers., 2012) ; en janvier 2013, deux autres nématodes ont été obtenus de frelons adultes dans un état de décomposition avancée, provenant d'un nid détruit à Issigeac, Dordogne (P. Doumenjou-Larroque. comm. pers., 2013). Ces parasites ont été envoyés au MNHN pour identification.

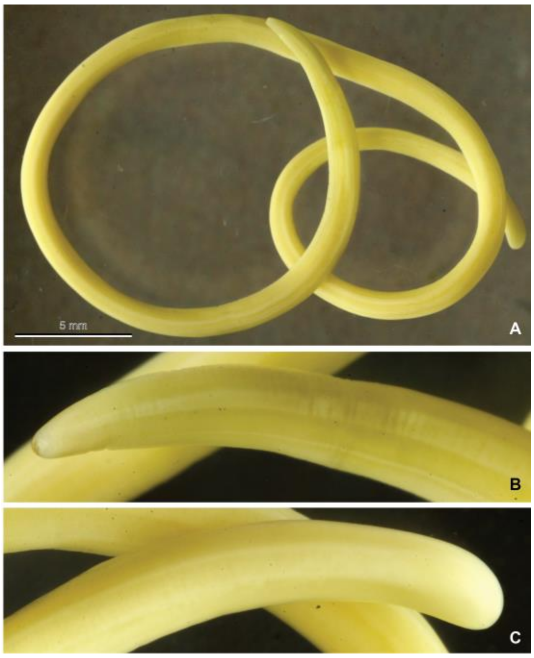

Figure 2. Photographie d'un des nématodes mermithidés étudiés (spécimen de Dompierre-sur-Bresbe - MNHN JL50). A : ver entier, B : Tête ; C : queue.

Morphologie
Le mermithidé de Dompierre-sur-Besbre (Allier) a été photographié (Figure 2) et mesuré (longueur, largeur), puis une partie du ver a été prélevée pour étude moléculaire tandis que le reste était envoyé à l'un des auteurs (GOP) pour une identification plus poussée. A maturité, la taille des nématodes mermithidés, y compris l'espèce concernée par cette étude, dépasse souvent largement celle de leur hôte. Le spécimen extrait du frelon asiatique est un juvénile en phase postparasitiaire dont les caractères morphologiques sont ceux du genre *Pheromermis* Poinar, Lane et Thomas, 1976. Les échantillons ont été déposés dans la collection du MNHN sous les références suivantes : MNHN JL50 (Dompierre-sur-Besbre) et MNHN JL51A et JL51B (Issigeac).

Identification moléculaire
L'ADN génomique total a été extrait à partir d'un segment de 5 mm de chacun des individus, à l'aide du kit Qiagen DNA Mini et du protocole fourni par le fabricant. Trois gènes candidats ont été sélectionnés pour l'amplification par PCR : un gène mitochondrial, la cytochrome oxydase (COI), et des gènes nucléaires, la grande et la petite sous-unité de l'ARN ribosomal (28S-rRNA et 18S-rRNA). Ce choix devait permettre de faire des comparaisons avec le grand nombre de séquences de nématodes déjà disponibles dans GenBank.

Les gènes ont été amplifiés en utilisant des amorces et des profils d'amplification standards (Tableau 1). Les PCR ont été effectuées dans un volume de réaction de 20 µl, avec 1 à 5 ng d'ADN et à une concentration finale de 1X tampon réactionnel, 2,5 mM MgCl2, 0,26 nM dNTP, 0,3 µM de chaque amorce, 5 % de DMSO et 1,5 unités de Qiagen Taq polymérase. Pour toutes les combinaisons de paires d'amorces, le profil d'amplification était le suivant : 5 min de dénaturation initiale à 94 °C, 40 cycles de 40 s à 94 °C, 40 s à la température d'hybridation des amorces (cf. Tableau 1) et 60 s à 72 °C, suivis d'une extension finale de 5 min à 72 °C. Les produits de PCR ont été révélés sur un gel d'agarose à 1,5 % coloré au bromure d'éthidium et les PCR positives ont été séquencées dans les deux directions à l'aide de la méthode de Sanger.

Tableau 1. Paires d'amorces utilisées dans cette étude avec leurs températures d'hybridation. Le 18S a été amplifié en deux fragments chevauchants.

Gène	Amorce		T d'hybridation	Référence
18S	18S-1F	TACCTGGTTGATCCTGCCAGTAG	51	Giribet et al. 1996
	18S-5R	CTTGCAAAGCTGCTTTCGC		
	18S-3F	GTTCGATTCCGGAGAGGGA	51	
	18S-Bi	GAGTCTCGTTCGTTATCGGA		
28S	28S-C1	ACCCGCTGAATTTAAGCAT	55	Dayrat et al 2001
	28S-D2	TCCGTGTTTCAAGACGGG		
COI	AnCO1-F	ATTTGGTCTTTGATCTGGTATGG	48	Cross et al 2006
	AnCO1-R	TGGCAGAAATAACATCCAAACTAG		
	LCO1490	GGTCAACAAATCATAAAGATATTGG	48	Folmer et al. 1994
	HCO2198	TAAACTTCAGGGTGACCAAAAAATCA		

Une recherche BLAST préliminaire suggère que les séquences 18S et 28S obtenues à partir des échantillons étaient similaires à celles des Mermithidae présents dans GenBank. Toutefois, seulement deux des douze séquences 28S enregistrées dans GenBank chevauchaient de façon significative les séquences obtenues ; c'est pourquoi les autres comparaisons ont été limitées au gène 18S. Toutes les séquences 18S de Mermithidae disponibles dans GenBank ont été téléchargées et alignées avec les séquences obtenues dans cette étude, ce qui a abouti à un alignement de 1322 pb. Les séquences 18S disponibles dans GenBank étaient de longueur variable, et certaines présentaient un chevauchement minimal avec la région amplifiée à partir de nos amorces. Le jeu de données a donc été réduit, pour ne retenir que les séquences de plus de 400 pb. Les nouvelles séquences ont été enregistrées dans GenBank sous les numéros KR029620 and KR029622 (MNHN JL50) and KR029621 and KR029623 (MNHN JL51A).

La matrice finale, qui inclut 26 séquences pour le groupe interne (Mermithidae) et cinq pour le groupe externe (Aulolaimidae : *Aulolaimus* et Isolaimiidae : *Isolaimium*) a été analysée selon le critère de maximum de vraisemblance, avec RAxML v. 7.0.3 (Stamatakis, 2006), en sélectionnant un modèle GTR+Γ+I et un arbre initial aléatoire, avec des fréquences de base empiriques, des paramètres α-shape et GTR-rates. Le support des nœuds a été estimé à partir d'un bootstrap de 100 réplicats.

RESULTATS

Morphologie

Seul le spécimen de Dompierre-sur-Besbre (un juvénile de 81 mm de long et 1,3 mm de diamètre) a pu être étudié d'un point de vue morphologique (Figure 2), les deux spécimens d'Issigeac étant en effet trop endommagés par la putréfaction. Les caractéristiques de cet unique spécimen sont compatibles avec celles d'un juvénile de *Pheromermis vesparum* Kaiser, 1987, un mermithidé parasite de guêpes sociales européennes.

Le genre *Pheromermis* se caractérise par : quatre papilles céphaliques submédianes, des amphides antérieures grandes et en forme de coupe, un vagin en forme de S non replié transversalement par rapport au corps, six cordons hypodermiques, des spicules pairs, courts et distincts, une cuticule avec des fibres transversales et des œufs dépourvus de processus (Poinar, Lane et Thomas, 1976). Cependant, ces caractères sont ceux des adultes de *Pheromermis* alors que les individus provenant du frelon asiatique sont des juvéniles postparasitaires ; il est donc possible que tous les spécimens étudiés n'appartiennent pas à la même espèce de *Pheromermis*.

Identification moléculaire

Parmi les trois spécimens analysés, l'un des individus d'Issigeac était probablement trop décomposé et son ADN trop dégradé pour que l'amplification ait lieu. Par contre, les gènes 18S et 28S des deux autres spécimens provenant de localités distantes ont pu être séquencés. Les séquences 18S des deux spécimens sont identiques, alors que celles du 28S ne diffèrent que de 1,6 %, toutes les positions variables étant concentrées dans les boucles. Malgré plusieurs tentatives, l'amplification du gène COI a échoué, ce qui suggère que les amorces standards ne permettent pas d'amplifier le gène COI de cette espèce.

Dans l'arbre obtenu par maximum de vraisemblance, les deux séquences 18S de nos spécimens les placent dans la famille des Mermithidae (Fig. 3) et comme espèces-sœurs de trois spécimens identifiés comme *Mermis nigrescens*, un parasite de sauterelles (Baker & Capinera, 1997). Ces résultats confortent l'identification morphologique, bien qu'aucune donnée moléculaire de *Pheromermis* ne soit actuellement disponible pour comparaison. Ne pouvant identifier ces spécimens à l'espèce, nous les considérons comme des *Pheromermis* sp. Toutefois, les juvéniles infestant *V. velutina* appartiennent très probablement à *Pheromermis vesparum* Kaiser, 1987, un parasite bien connu des guêpes sociales. Il a été signalé en effet comme parasite de *Vespa crabro, Vespula vulgaris, V. germanica, Dolichovespula saxonica* et *Polistes* sp. (Kaiser, 1987).

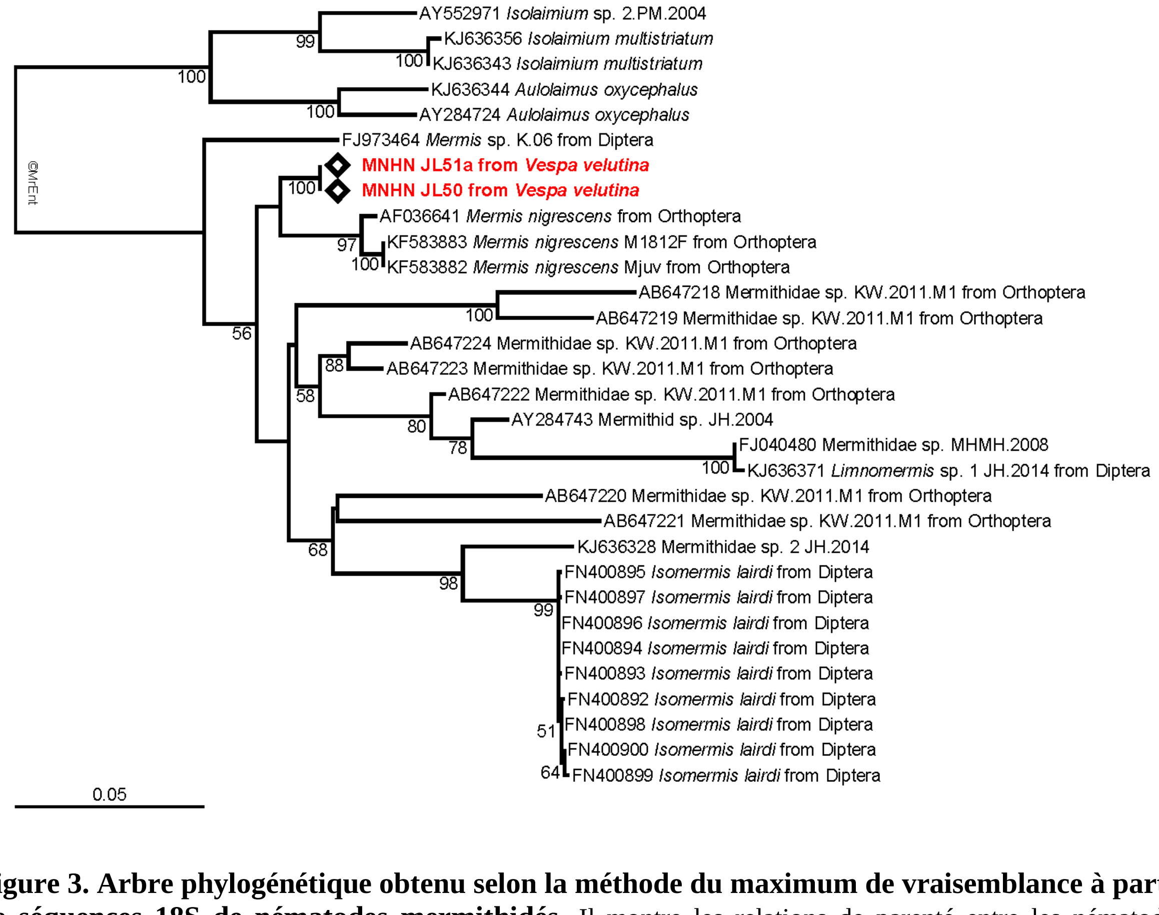

Figure 3. Arbre phylogénétique obtenu selon la méthode du maximum de vraisemblance à partir de séquences 18S de nématodes mermithidés. Il montre les relations de parenté entre les nématodes parasites infectant *Vespa velutina* (en rouge) et d'autres espèces de mermithidés. Lorsqu'ils sont connus, les insectes hôtes sont indiqués après le nom de chaque parasite. Les valeurs de bootstrap sont indiquées au niveau des nœuds.

DISCUSSION

En dix ans, seulement 3 nématodes ont été obtenus de frelons collectés dans deux localités françaises relativement éloignées. Or, les colonies de *V. velutina* sont très populeuses et chaque année un très grand nombre de nids sont détruits, puis détachés des hautes branches des arbres de sorte que leur contenu se répand sur le sol ; nombreux sont les frelons morts qui sont écrasés au cours de l'opération. Pourtant malgré le nombre considérable de nids détruits et de frelons manipulés, seuls un très petit nombre de nématodes parasites ont été observés, malgré leur grande taille (près de 10 cm). Ceci suggère que l'infection de *V. velutina* par ces nématodes demeure exceptionnelle.

Le cycle de développement des *Pheromermis* est unique au sein des Mermithidae car il implique l'intervention d'un hôte secondaire, appelé hôte paraténique ou hôte de transport (Poinar, Lane & Thomas, 1976 ; Kaiser, 1987 ; Martin, 2004). Les nématodes adultes pondent dans l'eau ou dans les sols saturés en eau des œufs dont l'embryon a déjà achevé son développement. Les œufs éclosent dans le tube digestif de divers insectes aquatiques ou semi-aquatiques puis les stades juvéniles infectieux du nématode traversent l'épithélium digestif de leur hôte pour entrer en quiescence dans ses tissus périphériques, même si l'hôte est en train de subir sa métamorphose imaginale. Les larves de frelons sont infectées lorsqu'elles sont nourries avec la chair des hôtes paraténiques capturés par des ouvrières. Les larves de nématodes reprennent alors leur activité et consomment les tissus non vitaux des larves de frelons en cours de croissance. L'infection par les nématodes coïncide chez les guêpes sociales avec la période de développement de la génération sexuée (Kaiser, 1987). L'hôte infecté est rarement tué avant sa métamorphose de sorte que, ayant atteint le stade adulte, il renferme dans son abdomen un long ver proche de la maturité, ce qui le rend stérile ou sexuellement inactif. Dès que le frelon parasité s'approche de l'eau en automne (avant que les futures reines n'entrent en hivernation), le nématode quitte son hôte et le tue, puis il subit sa mue imaginale et boucle son cycle en s'accouplant et déposant ses œufs dans l'eau. On ne sait pas encore si les frelons sexués adultes s'approchent naturellement des points d'eau ou si, au contraire, c'est le fait d'être parasités par un nématode qui les conduit à se jeter à l'eau (Poinar, 1976).

Deux hypothèses peuvent être évoquées pour expliquer la présence de nématodes parasites chez des adultes de *V. velutina*. Le parasite (1) peut avoir été introduit dans l'aire d'invasion par l'envahisseur lui-même, ou (2) peut provenir de la faune locale. La première hypothèse est peu vraisemblable car si des reines de frelons parasitées avaient été introduites, elles seraient mortes avant d'avoir produit des descendants. Une étude génétique récente (Arca et al., 2015) a par ailleurs montré que l'introduction de *V. velutina* en France s'est faite à partir d'un très petit nombre de reines, voire même d'une seule, ce qui rend encore moins probable l'idée qu'un parasite exotique ait pu être introduit avec son hôte. Il aurait fallu en outre que ce parasite soit capable de s'adapter à une ou plusieurs espèces d'insectes aquatiques locales susceptible de jouer le rôle d'hôtes paraténiques. La seconde hypothèse semble la plus probable. L'espèce invasive a été infestée par un nématode autochtone dont les hôtes paraténiques sont divers insectes aquatiques autochtones et les hôtes principaux des guêpes sociales européennes. Les espèces de *Pheromermis* qui attaquent les guêpes sociales en Europe ont un large éventail d'hôtes (Molloy, Vinikour & Anderson, 1999) et sont donc plus aptes à infecter une nouvelle espèce de guêpe sociale que des nématodes dont le spectre d'hôtes est plus restreint.

Les hôtes paraténiques connus des *Pheromermis* parasites de guêpes sociales comprennent notamment des larves de trichoptères (Trichoptera), de perles (Plecoptera), de tipules (Diptera Tipulidae) et d'éphémères (Ephemeroptera), ainsi que diverses larves de

coléoptères (Poinar, Lane et Thomas, 1976 ; Poinar, 1981 ; Molloy, Vinikour & Anderson, 1999). Une étude en cours sur le régime alimentaire de *V. velutina* nous a permis de recueillir plus de 2 000 boulettes de proies au moment où elles sont rapportées au nid par les ouvrières. L'identification des proies a montré que, parmi les hôtes paraténiques potentiels du nématode, seuls les trichoptères sont des proies pour *V. velutina* et seulement en très faible proportion (0,2 %) par rapport aux autres insectes capturés (données non publiées des auteurs).

Le recrutement local d'ennemis naturels tels que les nématodes mermithidés conduit évidemment à poser la question suivante : ces parasites sont-ils en mesure de contrôler les populations du frelon ? Le fait que les *Pheromermis* tuent leurs hôtes, fait de ces nématodes d'important auxiliaires potentiels pour la lutte biologique contre les guêpes sociales. Cependant, Martin (2004) note que, contrairement au taux (50 %) évoqué par Poinar, Lane et Thomas (1976) et par Moller et al. (1991), les taux d'infection des colonies de guêpes sociales par *Pheromermis* spp. sont généralement plus faibles et varient de 0 à 7 % pour les ouvrières et les mâles, et de 8 à 35 % pour les futures reines (Blackith & Stevenson, 1958 ; Kaiser, 1987). Fox-Wilson (1946) signale par ailleurs qu'en 1893, tous les mâles d'un grand nid de *Vespula* sp. étaient infectés par les vers de *Gordius* sp. (les espèces de ce genre appartiennent aux Nematomorpha, un phylum distinct de celui des Nematoda dont font partie les Mermithidae, mais elles ont un cycle de développement similaire). Les dissections effectuées par différents chercheurs sur des milliers de guêpes adultes provenant de centaines de nids de *Vespula* et *Vespa* montrent cependant que de tels niveaux d'infection sont extrêmement rares (Martin, 2004). Notre étude portant sur 77 nids et 30 000 adultes (Rome et al., 2015) nous mène à la même conclusion.

Le niveau d'infection d'une colonie donnée dépend aussi de la distance séparant le nid de guêpes sociales d'une source d'hôtes paraténiques suffisamment abondante (Rose, Harris & Glare, 1999 ; Martin, 2004). Kaiser (1987) a constaté qu'un tiers des nids de *Vespula* spp. sont infectés par *Pheromermis* sp., quand et uniquement quand ces derniers étaient installés à moins de 200 m d'un point d'eau. La rareté des hôtes paraténiques potentiels dans le spectre de proies de *V. velutina* ne permet pas non plus au nématode d'infester fortement une colonie, même si une ouvrière de frelon nourrit généralement plusieurs larves avec la chair d'une seule boulette de proie rapportée au nid (Janet, 1903 ; Spradbery, 1973). Enfin, la mort de nombreux frelons n'est pas suffisante pour entraîner la destruction totale d'une colonie ; elle peut en effet survivre même après la disparition de 75 % de ses individus (Gambino, Pierluisi & Poinar, 1992 ; Toft & Harris, 2004 ; Gouge, 2005). La reine qui a fondé la colonie ne peut pas non plus être infectée puisque le nématode infeste son hôte à l'état larvaire et le tue lorsqu'il a atteint le stade adulte en automne ; si de futures reines sont infectées, elles meurent donc avant de fonder une colonie. Le nématode occupe à maturité tout l'abdomen de son hôte, ce qui limite considérablement la capacité des reines infectées à accumuler des réserves de graisse (Martin, 2004). Enfin, contrairement à de nombreux autres nématodes entomopathogènes, les *Pheromermis* spp. ne sont pas vecteurs de bactéries symbiotiques pathogènes pour les insectes (Poinar, 1979), dont la présence pourrait accroître la virulence du parasite (Lacey et al., 2001).

L'efficacité potentielle des *Pheromermis* spp. comme auxiliaires de lutte biologique contre les guêpes sociales a été testée à partir du modèle de Martin (2014) qui prédit que la production de sexués est réduite lorsque les colonies subissent précocement de forts niveaux d'infection. Cependant, même les colonies infectées à 80 % produisent encore quelques descendants sexués, ce qui montre qu'elles sont résilientes face à l'infection. De plus, l'augmentation du niveau d'infestation accroit aussi le rapport entre larves et ouvrières (les larves sont alors moins bien nourries) de sorte que les larves de la génération sexuée produisent des femelles de moins bonne qualité, qui sont donc moins aptes à hiverner et

fonder une nouvelle colonie (Harris & Beggs, 1995). Curieusement, la mauvaise qualité de certaines femelles reproductrices peut favoriser localement la fondation d'un plus grand nombre de colonies via un phénomène de compensation densité-dépendant. En effet, les femelles en bonne santé (seules capables de fonder un nid) sont moins nombreuses et meurent moins du fait de la compétition intraspécifique, que ce soit en automne lors de l'acquisition de réserves de graisse (Harris & Beggs, 1995) ou au printemps lors des tentatives répétées d'usurpation des nids (Spradbery, 1991 ; Martin, 1991, 2004 ; Archer, 2012).

La réduction de la compétition intraspécifique, qui provoque habituellement la mort d'un très grand nombre de fondatrices au printemps, explique aussi pourquoi les mouches Conopidae ne peuvent être des antagonistes efficaces des guêpes sociales, même si elles sont capables de parasiter directement les jeunes reines au printemps. Le fait que l'espèce autochthone *Conops vesicularis* puisse parasiter *Vespa velutina* en France (Darrouzet, Gévar & Dupont, 2014) n'en fait donc pas un auxiliaire de choix pour lutter contre le frelon invasif. De plus, les Conopidae volent surtout en été, entre juin et septembre (Schmid-Hempel et al., 1990) et s'attaquent donc plus aux ouvrières de frelon qu'aux jeunes reines car ces dernières ne quittent plus leur nid dès que leurs premières ouvrières ont émergé au printemps (Matsuura & Yamane, 1990 ; Rome et al., 2015).

A l'instar de *Pheromermis* spp., de nombreux parasitoïdes de guêpes sociales, comme les Ichneumonidae *Sphecophaga* spp. (Donovan et al., 2002 ; Beggs et al., 2008), les Stylopidae *Xenos* spp. (Matsuura & Yamane, 1990), ou des Conopidae tels que *C. vesicularis*, attaquent seulement des individus isolés au sein d'une colonie. En revanche, d'autres parasites tels que l'acarien *Varroa destructor* peuvent tuer une colonie d'abeilles mellifères de plus de 30 000 individus en transmettant des virus pathogènes qui se dispersent au sein de la ruche et infectent un très grand nombre d'abeilles (Sumpter & Martin, 2004). Dans le cas des parasites attaquant un hôte unique, le taux d'infection au sein de la colonie doit être très élevé (> 50 %) pour provoquer sa destruction ou réduire de manière significative sa productivité (Matsuura & Yamane, 1990 ; Barlow, Beggs et Barron, 2002), parce que les populations de guêpes sociales possèdent de grandes capacités de reproduction et une forte résilience due aux phénomènes de compensation densité-dépendants (Martin, 1991, 2004).

Au lieu de diluer les effets d'un parasite, la présence d'un hôte alternatif (et envahissant) peut créer un réservoir d'infection, d'autant plus important que l'hôte invasif est abondant. L'arrivée d'un nouvel hôte dans une région donnée peut ainsi favoriser la multiplication du parasite indigène, qui peut alors devenir plus dangereux pour des populations sensibles d'hôtes locaux (Holt & Lawton, 1993). Cette concurrence indirecte entre les hôtes d'un même ennemi naturel peut conduire à l'extinction de l'hôte le plus parasité (Prenter et al., 2004 ; Dunn, 2009). Les nématodes sont considérés comme peu nuisibles aux guêpes sociales (Gouge, 2005) alors que les mouches conopides, qui attaquent rarement les guêpes sociales (Spradbery, 1973 ; Matsuura & Yamane, 1990), peuvent par contre être localement très nuisibles aux colonies de bourdons en Europe (Schmid-Hempel 2001). Les effets négatifs des parasites sur leurs hôtes ne sont cependant pas toujours évidents ni immédiatement perceptibles ; ils dépendent souvent de l'état de santé de l'hôte et ne deviennent dommageables que lorsque la population de l'hôte est affaiblie (Schmid-Hempel, 2001), une situation qui concerne aujourd'hui davantage les bourdons, dont les populations sont en déclin (Gillespie, 2010), que les guêpes sociales.

REMERCIEMENTS

Nous sommes très reconnaissants aux personnes qui ont collecté ces nématodes : Ph. Noireterre et P. Doumenjou-Larroque, ainsi qu'à Claire Ménissier de la revue "La Hulotte" qui nous a mis en contact avec le second collecteur, et à toutes les personnes qui ont fourni des nids pour notre étude. Nous remercions également les deux relecteurs anonymes pour leurs précieux commentaires sur le manuscrit.

REFERENCES

Arca M, Mougel F, Guillemaud T, Dupas S, Rome Q, Perrard A, Muller F, Fossoud A, Capdevielle-Dulac C, Torres-Leguizamon M, Chen X-X, Tan J, Jung C, Villemant C, Arnold G, Silvain J-F. 2015. Reconstructing the invasion and the demographic history of the yellow-legged hornet, *Vespa velutina*, in Europe. *Biological Invasions* DOI 10.1007/s10530-015-0880-9.

Arca M, Papachristoforou A, Mougel F, Rortais A, Monceau K, Bonnard O, Tardy P, Thiéry D, Silvain J-F, Arnold G. 2014. Defensive behaviour of *Apis mellifera* against *Vespa velutina* in France: Testing whether European honeybees can develop an effective collective defence against a new predator. *Behavioural Processes* 106:122–129 DOI 10.1016/j.beproc.2014.05.002.

Archer ME. 2012. *Vespine wasps of the world. Behaviour, Ecology and Taxonomy of the Vespinae.* Manchester: Siri Scientific Press.

Baker GL, Capinera JL. 1997. Nematodes and nematomorphs as control agents of grasshoppers and locusts. *Memoirs of the Entomological Society of Canada* 129:157–211 DOI 10.4039/entm129171157-1.

Barbet-Massin M, Rome Q, Muller F, Perrard A, Villemant C, Jiguet F. 2013. Climate change increases the risk of invasion by the Yellow-legged hornet. *Biological Conservation* 157:4–10 DOI 10.1016/j.biocon.2012.09.015.

Barlow ND, Beggs JR, Barron MC. 2002. Dynamics of common wasps in New Zealand beech forests: a model with density dependence and weather. *Journal of Animal Ecology* 71:663–671 DOI 10.1046/j.1365-2656.2002.00630.x.

Beggs JR, Rees JS, Toft RJ, Dennis TE, Barlow ND. 2008. Evaluating the impact of a biological control parasitoid on invasive *Vespula* wasps in a natural forest ecosystem. *Biological control* 44:399–407 DOI 10.1016/j.biocontrol.2007.10.016.

Beggs JR, Brockerhoff EG, Corley JC, Kenis M, Masciocchi M, Muller F, Rome Q, Villemant C. 2011. Ecological effects and management of invasive alien Vespidae. *BioControl* 56:505–526 DOI 10.1007/s10526-011-9389-z.

Blackith RE, Stevenson JH. 1958. Autumnal populations of wasps nests. *Insectes sociaux* 5:347–352.

Colautti RI, Ricciardi A, Grigorovich IA, MacIsaac HJ. 2004. Is invasion success explained by the enemy release hypothesis? *Ecology Letters* 7:721–733 DOI 10.1111/j.1461-0248.2004.00616.x.

Cross MA, Collins C, Campbell N, Watts PC, Chubb JC, Cunningham CO, Hatfield EMC, MacKenzie K. 2006. Levels of intra-host and temporal sequence variation in a large CO1 sub-units from *Anisakis simplex* sensu stricto (Rudolphi 1809) (Nematoda: Anisakisdae): implications for fisheries management. *Marine Biology* 151:695–702 DOI 10.1007/s00227-006-0509-8.

Darrouzet E, Gévar J, Dupont S. 2014. A scientific note about a parasitoid that can parasitize the yellow-legged hornet, *Vespa velutina nigrithorax*, in Europe. *Apidologie* 46:130–132 DOI 10.1007/s13592-014-0297-y.

Dayrat B, Tillier A, Lecointre G, Tillier S. 2001. First molecular evidence for the existence of a Tardigrada + Arthropoda clade. *Molecular Phylogenetics and Evolution* 19:225–235 DOI 10.1006/mpev.2001.0926.

Demichelis S, Manino A, Minuto G, Mariotti M, Porporato M. 2014. Social wasp trapping in north west Italy: comparison of different bait-traps and first detection of *Vespa velutina*. *Bulletin of Insectology* 67:307–317.

Donovan BJ, Havron A, Leathwick DM, Ishay JS. 2002. Release of *Sphecophaga orientalis* Donovan (Hymenoptera: Ichneumonidae: Cryptinae) in New Zealand as a possible "new association" biocontrol agent for the adventice social wasps *Vespula germanica* (F.) and *Vespula vulgaris* (L.) (Hymenoptera: Vespidae: Vespinae). *New Zealand Entomologist* 25:17–25 DOI 10.1080/00779962.2002.9722090.

Dunn AM. 2009. Chapter 7 Parasites and Biological Invasions. In: Joanne P. Webster ed. *Advances in Parasitology*. Natural History of Host-Parasite Interactions. Academic Press, 161–184.

Folmer O, Black M, Hoeh W, Lutz R, Vrijenhoek R. 1994. DNA primers for amplification of mitochondrial cytochrome c oxidase subunit I from diverse metazoan invertebrates. *Molecular Marine Biology and Biotechnology* 3:294–299.

Fox-Wilson G. 1946. Factors affecting populations of social wasps, *Vespula* species, in England (Hymenoptera). In: *Proceedings of the Royal Entomological Society of London. Series A, General Entomology*. Wiley Online Library, 17–27.

Gambino P, Pierluisi GJ, Poinar GOJ. 1992. Field test of the nematode *Steinernema feltiae* (Nematoda: Steinernematidae) against yellowjacket colonies (Hym.: Vespidae). *Entomophaga* 37:107–114 DOI 10.1007/BF02372979.

Gillespie S. 2010. Factors affecting parasite prevalence among wild bumblebees. *Ecological Entomology* 35:737–747 DOI 10.1111/j.1365-2311.2010.01234.x.

Girardoz S, Kenis M, Quicke DLJ. 2006. Recruitment of native parasitoids by an exotic leaf miner, *Cameraria ohridella*: host-parasitoid synchronization and influence of the environment. *Agricultural and Forest Entomology* 8:49–56 DOI 10.1111/j.1461-9555.2006.00281.x.

Giribet G, Carranza S, Baguna J, Ruitort M, Ribera C. 1996. First molecular evidence for the existence of a Tardigrada + Arthropoda clade. *Molecular Biology and Evolution* 13:76–84 DOI 10.1093/oxfordjournals.molbev.a025573.

Gouge DH. 2005. Application for social insect control. In: *Nematods as biocontrol agents*. Grewal PS, Ehlers RU, Shapiro-Ilan DI, Wallingford, UK: CABI Publishing, 317–329.

Harris RJ, Beggs J. 1995. Variation in the quality of *Vespula vulgaris* (L.) queens (Hymenoptera: Vespidae) and its significance in wasp population dynamics. *New Zealand Journal of Zoology* 22:131–142 DOI 10.1080/03014223.1995.9518030.

Holt RD, Lawton JH. 1993. Apparent competition and enemy-free space in insect host-parasitoid communities. *American Naturalist* 142:623–645 DOI 10.1086/285561.

Holway DA, Suarez AV, Case TJ. 1998. Loss of Intraspecific Aggression in the Success of a Widespread Invasive Social Insect. *Science* 282:949–952 DOI 10.1126/science.282.5390.949.

INPN. 2015. *Vespa velutina* Lepeletier, 1836. *Available at http://inpn.mnhn.fr/espece/cd_nom/433589* (accessed 19 February 2015).

Janet C. 1903. *Observations sur les guêpes*. Paris: C. Naud.

Kaiser H. 1987. Biologie, Okologie und Entwicklung des europaischen Wespen-Parasitoiden *Pheromermis vesparum* n. sp. (Mermithidae, Nematoda). *Zoologische Jahrbucher. Abteilung fur Systematik, Okologie und Geographie der Tiere*.

Kenis M, Auger-Rozenberg M-A, Roques A, Timms L, Péré C, Cock MJW, Settele J, Augustin S, Lopez-Vaamonde C. 2009. Ecological effects of invasive alien insects. In: Langor DW, Sweeney J eds. *Ecological Impacts of Non-Native Invertebrates and Fungi on Terrestrial Ecosystems*. Springer Netherlands, 21–45.

Lacey LA, Frutos R, Kaya HK, Vail P. 2001. Insect Pathogens as Biological Control Agents: Do They Have a Future? *Biological Control* 21:230–248 DOI 10.1006/bcon.2001.0938.

Lee KA, Klasing KC. 2004. A role for immunology in invasion biology. *Trends in Ecology & Evolution* 19:523–529 DOI 10.1016/j.tree.2004.07.012.

Martin S. 1991. A Simulation Model for Colony Development of the Hornet *Vespa simillima* (Hymenoptera, Vespidae). *Japanese journal of entomology* 59:105–124.

Martin SJ. 2004. A simulation model of biological control of social wasps (Vespinae) using mermithid nematodes. *New Zealand Journal of Zoology* 31:241–248 DOI 10.1080/03014223.2004.9518376.

Matsuura M, Yamane S. 1990. *Biology of the Vespine Wasps*. Berlin; New York: Springer-Verlag.

Moller H, Clapperton BK, Alspach PA, Tilley JAV. 1991. Comparative seasonality of *Vespula germanica* (F.) and *Vespula vulgaris* (L.) colonies (Hymenoptera: Vespidae) in urban Nelson, New Zealand. *New Zealand Journal of Zoology* 18:111–120 DOI 10.1080/03014223.1991.10757957.

Molloy DP, Vinikour WS, Anderson RV. 1999. New North American Records of Aquatic Insects as Paratenic Hosts of *Pheromermis* (Nematoda: Mermithidae). *Journal of Invertebrate Pathology* 74:89–95 DOI 10.1006/jipa.1999.4860.

Monceau K, Arca M, Leprêtre L, Mougel F, Bonnard O, Silvain J-F, Maher N, Arnold G, Thiéry D. 2013. Native Prey and Invasive Predator Patterns of Foraging Activity: The Case of the Yellow-Legged Hornet Predation at European Honeybee Hives. *PLoS ONE* 8:e66492 DOI 10.1371/journal.pone.0066492.

Péré C, Bell R, Turlings TCJ, Kenis M. 2011. Does the invasive horse-chestnut leaf mining moth, *Cameraria ohridella*, affect the native beech leaf mining weevil, *Orchestes fagi*, through apparent competition? *Biodiversity and Conservation* 20:3003–3016 DOI 10.1007/s10531-011-0134-9.

Perrard A, Haxaire J, Rortais A, Villemant C. 2009. Observations on the colony activity of the Asian Hornet *Vespa velutina* Lepeletier 1836 (hymenoptera: Vespidae: Vespinae) in France. *Annales de la Société Entomologique de France* 45:119-127. DOI 10.1080/00379271.2009.10697595.

Poinar GO. 1976. Presence of Mermithidae (Nematoda) in Invertebrate Paratenic Hosts. *The Journal of Parasitology* 62:843-844 DOI 10.2307/3278981.

Poinar GO Jr. 1979. *Nematodes for biological control of insects*. Boca Raton, Florida, USA: CRC Press, Inc.

Poinar GOJ. 1981. Distribution of *Pheromermis pachysoma* (Mermithidae) Determined by Paratenic Invertebrate Hosts. *Journal of Nematology* 13:421–426.

Poinar GO, Lane RS, Thomas GM. 1976. Biology and Redescription of *Pheromermis pachysoma* (V. Linstow) N. Gen., N. Comb. (Nematoda: Mermithidae), a Parasite of Yellowjackets (Hymenoptera: Vespidae). *Nematologica* 22:360–370a DOI 10.1163/187529276X00652.

Prenter J, MacNeil C, Dick JTA, Dunn AM. 2004. Roles of parasites in animal invasions. *Trends in Ecology & Evolution* 19:385–390 DOI 10.1016/j.tree.2004.05.002.

Rasplus J-Y, Villemant C, Paiva MR, Delvare G, Roques A. 2010. Hymenoptera. Chapter 12. In: Roques A ed. *Arthropod invasions in Europe*. BioRisk, 669–776.

Rome Q, Dambrine L, Onate C, Muller F, Villemant C, García Pérez AL, Maia M, Carvalho Esteves P, Bruneau E. 2013. Spread of the invasive hornet *Vespa velutina* Lepeletier, 1836, in Europe in 2012 (Hym., Vespidae). *Bulletin de la Société entomologique de France* 118:21–22 inpn.mnhn.fr/docs/Vespa_velutina/1643-1-Rome_et_al_2013_p.pdf.

Rome Q, Muller FJ, Touret-Alby A, Darrouzet E, Perrard A, Villemant C. 2015. Caste differentiation and seasonal changes in *Vespa velutina* (Hym.: Vespidae) colonies in its introduced range. *Journal of Applied Entomology*: DOI 10.1111/jen.12210.

Rortais A, Villemant C, Gargominy O, Rome Q, Haxaire J, Papachristoforou A, Arnold G. 2010. A New Enemy of Honeybees in Europe: The Asian Hornet *Vespa velutina*. In: Settele J ed. *Atlas of biodiversity risks – from Europe to the globe, from stories to maps*. Sofia, Moscow: Pensoft, 181.

Rose EAF, Harris RJ, Glare TR. 1999. Possible pathogens of social wasps (Hymenoptera: Vespidae) and their potential as biological control agents. *New Zealand Journal of Zoology* 26:179–190 DOI 10.1080/03014223.1999.9518188.

Roy HE, Handley L-JL, Schönrogge K, Poland RL, Purse BV. 2011. Can the enemy release hypothesis explain the success of invasive alien predators and parasitoids? *BioControl* 56:451–468 DOI 10.1007/s10526-011-9349-7.

Schmid-Hempel P, Müller C, Schmid-Hempel R, Shykoff JA. 1990. Frequency and ecological correlates of parasitism by conopid flies (Conopidae, Diptera) in populations of bumblebees. *Insectes Sociaux* 37:14–30 DOI 10.1007/BF02223812.

Schmid-Hempel P. 2001. On the evolutionary ecology of host–parasite interactions: addressing the question with regard to bumblebees and their parasites. *Naturwissenschaften* 88:147–158 DOI 10.1007/s001140100222.

Spradbery JP. 1973. *Wasps: An Account of the Biology and Natural History of Solitary and Social Wasps with Particular Reference to Those of the British Isles*. London: Sidgwick & Jackson.

Spradbery JP. 1991. An orphaned colony of the European wasp *Vespula germanica* (F.) (Hymenoptera: Vespidae) in Australia resulting from repeated usurpation. *New Zealand Journal of Zoology* 18:101–103 DOI 10.1080/03014223.1991.10757955.

Stamatakis A. 2006. RAxML-VI-HPC: maximum likelihood-based phylogenetic analyses with thousands of taxa and mixed models. *Bioinformatics* 22:2688–2690 DOI 10.1093/bioinformatics/btl446.

Sumpter DJT, Martin SJ. 2004. The dynamics of virus epidemics in *Varroa*-infested honey bee colonies. *Journal of Animal Ecology* 73:51–63 DOI 10.1111/j.1365-2656.2004.00776.x.

Toft RJ, Harris RJ. 2004. Can Trapping control Asia paper wasp (*Polistes chinensis antennalis*) populations? *New Zealand Journal of Ecology* 28:279–282.

Torchin ME, Mitchell CE. 2004. Parasites, Pathogens, and Invasions by Plants and Animals. *Frontiers in Ecology and the Environment* 2:183–190 DOI 10.2307/3868313.

Villemant C, Barbet-Massin M, Perrard A, Muller F, Gargominy O, Jiguet F, Rome Q. 2011. Predicting the invasion risk by the alien bee-hawking yellow-legged hornet *Vespa velutina nigrithorax* across Europe and other continents with niche models. *Biological Conservation* 144:2142–2150 DOI 10.1016/j.biocon.2011.04.009.

Villemant C, Rome Q, Muller F, Arca M, Maher N, Darrouzet E. 2008. *Étude de la biologie du comportement et de l'impact de Vespa velutina sur les abeilles en vue d'un contrôle spécifique*. Programme Communautaire pour l'Apiculture, CE n°797/2007-2010, rapport intermédiaire 2007-2008, 46 p
